# Rare complication of subacute renal artery intramural haematoma after renal artery stenting: a case report

**DOI:** 10.1093/ehjcr/ytz014

**Published:** 2019-04-08

**Authors:** Yang Chen, Hui Dong, Xiongjing Jiang, Wuqiang Che

**Affiliations:** Department of Cardiology, Fuwai Hospital, National Center for Cardiovascular Diseases, Chinese Academy of Medical Sciences and Peking Union Medical College, No. 167, Beilishi Road, Xicheng District, Beijing, China

**Keywords:** Renovascular hypertension, Renal artery stent, Intramural haematoma (IMH), Intravascular ultrasound (IVUS), Case report

## Abstract

**Background:**

Renal artery intramural haematoma (IMH) is a rare cause of renal artery obstruction after stenting. Diagnosis and treatment are difficult as there are only a few cases reported.

**Case summary:**

We present the case of sudden-onset abdominal pain and non-functional kidney 3 days after renal artery stent implantation. Subacute luminal narrowing of the renal artery was initially diagnosed using computed tomography angiography and renal artery angiography, and a final diagnosis of subacute renal artery IMH was made using intravascular ultrasound (IVUS). Subsequently, the patient was treated with percutaneous transluminal angioplasty from far to near and another stent implantation. At the third month follow-up, blood pressure and renal function were normal.

**Discussion:**

This case suggests that IVUS could be useful for qualifying and treating the subacute renal artery IMH.

**Learning points**

- Renal artery intramural haematoma (IMH) is a rare cause of renal artery obstructed after stenting.
- Intravascular ultrasound could be useful for qualifying and treating renal artery IMH.
- Percutaneous transluminal angioplasty from distal to proximal and another stent implantation is an important strategy for treating IMH.

## Introduction

Stent implantation is a treatment for renovascular hypertension due to atherosclerotic renal artery stenosis.^1^ Renal artery intramural haematoma (IMH) is a rare complication after renal artery stenting.^2^ It can be diagnosed using intravascular ultrasound (IVUS) instead of computed tomography angiography (CTA) and renal artery angiography (RAG). If severe renal artery IMH causes a non-functional kidney, intervention may be indicated. Balloon dilatation or stent closure is known to be more successful for managing severe renal artery IMH.

## Timeline

**Table:** 

Day	Hour	Events
0	09:00	Right renal artery stent successfully
3	06:58	Sudden right lower abdominal pain
3	09:00	Ultrasound (US): the main trunk of the right renal artery was not clear, and the blood flow was significantly reduced
3	10:00	Computed tomography angiography: the right renal artery stent was unobstructed, but the middle segment was severely obstructed, and the right renal perfusion was light
3	15:00	Dual glomerular filtration rate (GFR) examination discovered that the right kidney was non-functional (GFR: right 0.6; left 75.6)
4	09:00	Renal artery angiography: the right renal artery stent was patent and severe new stenosis of the middle part of the renal artery with two branches involved; however, there was no dissection or thrombus formation
4	09:10	IVUS: the IMH originated at the distal end of the stent, without an identifiable entry point
4	09:20	Percutaneous transluminal angioplasty form far to near and another stent implantation
30		Follow-up: the abdominal pain symptoms had disappeared, and the blood pressure and renal function were normal
90		US: the right renal artery was unobstructed

## Case presentation

**Figure 1:**
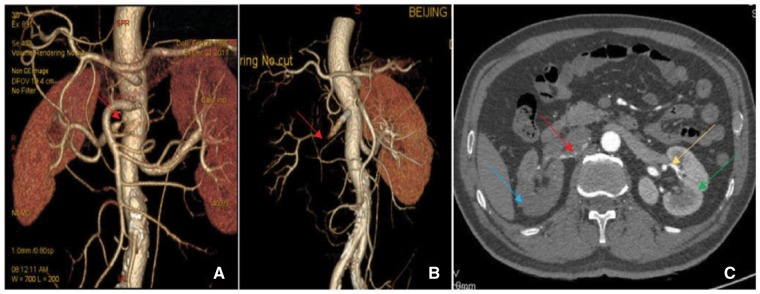
Preoperative renal artery computed tomography angiography shows that the right renal artery stenosis (red arrow) was 95% (*A*). Two days after renal artery stenting, reconstruction of renal artery computed tomography angiography showed the lumen of the middle part of the right renal artery was occluded (red arrow) and right renal perfusion was not restored (*B*). The transverse section highlights a long linear filling defect of the right renal artery (red arrow) with full filling of the left renal artery (yellow arrow) and no filling with contrast media in the right renal cortex (blue arrow) with full filling with contrast media in the left renal cortex (green arrow) (*C*).

**Figure 2:**
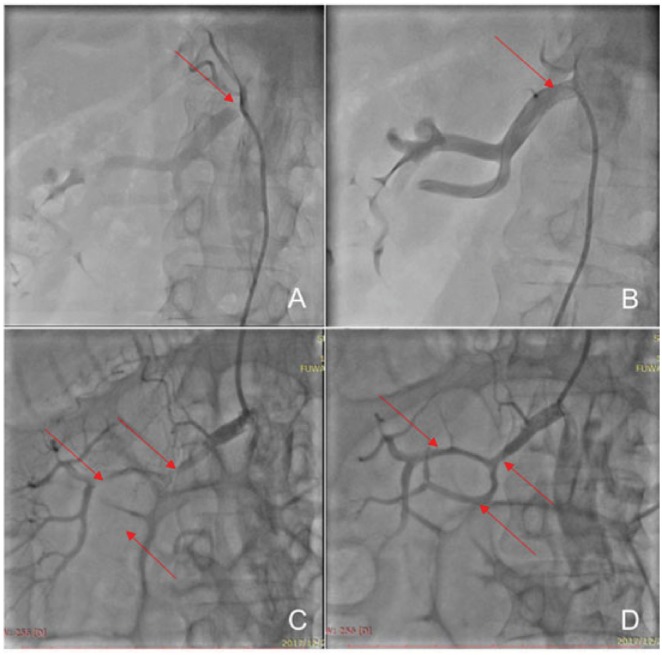
Renal artery angiography shows that the right renal artery stenosis (red arrow) was 95% (*A*). The right renal artery stent is perfect (red arrow), without any complications (*B*). The obstructed renal artery (red arrow) near the first stent (*C*). The patency of the right renal artery (red arrow) after PTA and stent re-implantation (*D*).

A 62-year-old man, a heavy smoker, was admitted with resistant hypertension. He had been a known hypertensive for >10 years; the highest systolic and diastolic blood pressure was 220 and 140 mmHg, respectively. Treatment with oral calcium channel blocker, beta blockers, and diuretics had not controlled the blood pressure. Other medical history included new-onset diabetes for the last 2 months and hyperlipidaemia and coronary heart disease for the last 10 years. No abdominal and cardiovascular abnormalities were found. Conn’s syndrome was excluded by negative aldosterone screening in horizontal position and no abnormal adrenal CT scan. The creatinine level was 84.3 µmol/L (44–133 µmol/L). Renal artery US showed a peak systolic velocity of 358 cm/s, indicating severe stenosis of the right renal artery. Renal artery CTA showed local and ostial stenosis (95%) of the right renal artery (*Figure 1A*). RAG showed 95% ostial stenosis of the right renal artery (*Figure 2A*) using a 6 F RDC catheter. The stenosis was pre-dilated with a 4 × 20 mm balloon (Sapphire) at a maximal pressure of 18 atm and implanted with a 6 × 14 mm stent (Express SD) at a maximal pressure of 15 atm through a Fielder guided wire. The intervention was successful without any complications (*Figure 2B,*Supplementary material online, *Video S1*). Dual antiplatelet therapy was then administered (aspirin 100 mg, one time daily, clopidogrel 75 mg, one time daily).

**Figure 3:**
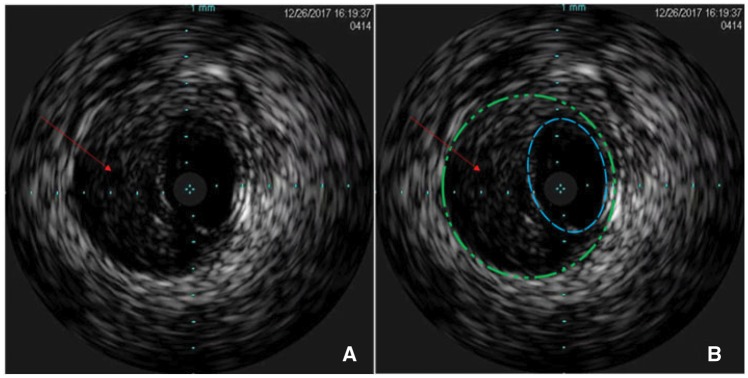
The original images of intravascular ultrasound (*A*). The schematic images of intravascular ultrasound shows a strong pulsatile echo area (red arrow) between the intima (blue line) and media (green line) of the right renal artery, resulting in the compression of the true lumen, and no obvious entry and exit of the interlayer and haematoma (*B*).

**Figure 4:**
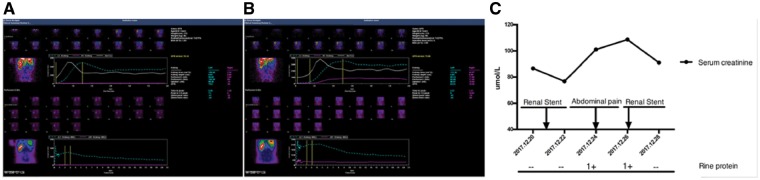
Dual glomerular filtration rate was measured 4 days after the first renal artery stenting, which revealed that the right kidney was non-functional (*A*). Dual glomerular filtration rate was measured 2 days after the second renal artery stenting, which revealed that the right kidney function had partially recovered. (*B*). The serum creatinine and urine protein of the patient were normal before the operation and there was no abnormality after primary renal artery stenting. Creatinine increased, and urine protein was weakly positive when the patient had sudden abdominal pain, but both values returned to normal after renal artery re-intervention (*C*).

After 3 days, the patient developed sudden right lower abdominal pain. The abdominal and renal examination was negative, but defaecation had stopped. The electrocardiogram, myocardial enzyme levels, and myocardial infarction marker levels were all normal. Pancreatitis was excluded because the amylase levels were normal. The serum creatinine level increased slightly, to 100–108 µmol/L, the urine protein was weakly positive (+1), and the blood pressure was maintained at 110/70 mmHg. To determine the cause of abdominal pain, renal artery US was performed, which showed that the main trunk of the right renal artery was not clear, and the blood flow was significantly reduced. This indicated a possible complication of right renal artery stenting. Renal artery CTA showed an unobstructed right renal artery stent, a severely stenosed middle segment, and light right renal perfusion (*Figure 1B *and* C*, Supplementary material online, *Video S2*). Considering a diagnosis of thrombosis or dissection of the renal artery, treatment with low-molecular weight heparin anticoagulant and rehydration was administered. Simultaneously, examining the dual glomerular filtration rate (GFR) using nuclear imaging discovered a non-functional right kidney (GFR: right 0.6; left 75.6; *Figure 4A*). Therefore, RAG was repeated to identify the cause of blood flow reduction and rescuing kidney function; the right renal artery stent was found patent. Severe stenosis (90–95%) of the middle part of the renal artery with two branches involved was seen; however, there was no dissection or thrombus formation (*Figure 2C*, Supplementary material online, *Video S3*). To further determine the aetiology of the new lesion, IVUS was used, which showed that the IMH originated at the distal end of the stent, without an identifiable entry point, and the length was about 40 mm (*Figure 3*, Supplementary material online, *Video S4*).

After clarifying the cause of the new stenosis, we first used a 2.5 × 20 mm balloon, followed by a 4 × 20 mm balloon (Sapphire) at a low pressure to push out the haematoma and dilate the compressed renal artery from the proximal to the distal end. The cavity of the compressed renal artery increased gradually, and the blood flow improved. However, the residual stenosis was still more than 50%, limiting the blood flow. Therefore, another stent (5 × 19 mm Express SD) was implanted near the first stent; the distal segment of the renal artery was seen to be well-developed (*Figure 2D*, Supplementary material online, *Video S5*). After reoperation, dual GFR revealed that the right kidney function had partially recovered (GFR: right 10.3; left 66.3; *Figure 4A*), the creatinine reduced to 91 µmol/L, and the urine protein was negative (*Figure 4C*). After discharge, the patient continued to maintain dual antiplatelet therapy. At the third month follow-up, the abdominal pain symptoms had disappeared, the serum creatinine level was 79 µmol/L, the urine protein was negative, and the blood pressure was maintained at 125/83 mmHg without any antihypertension drug. Renal artery US showed that the right renal artery was unobstructed.

## Discussion

Stent implantation is a treatment for renovascular hypertension due to atherosclerotic renal artery stenosis.^1^ Rare complications^3^^,^^4^ such as renal capsule haemorrhage and acute aortic IMH are reported. However, IMH of the renal artery is an extremely rare complication; there is only one report on renal artery IMH immediately caused by a cover stent that was used for treating renal artery dissection after the first renal artery stent implantation.^2^ In this case, IMH after renal artery stenting occurred late because the first stent was likely oversized; therefore, we report the rare complication of subacute IMH after renal artery stenting.

Numerous studies^5^^,^^6^ have reported on coronary artery and aorta IMH after stent implantation. Arteriography is sometimes inadequate in diagnosing IMH. IVUS could observe the location of the IMH, detect interlayer fractures, and determine the reason for the narrowing vascular lumen.^7^ Due to the lack of outlet, IMH can quickly enlarge, and severe compression of the lumen could cause coronary artery stenosis. Therefore, once coronary artery IMH is diagnosed, emergency treatment must be implemented,^8^ a cutting balloon dilatation to promote the transformation of typical dissection^9^ or stent closure.^10^ The pressure should be expanded properly from the distal to the proximal end to avoid expansion of the IMH,^7^ which is called a milking sandwich.^6^

In this case, the obstructed renal artery after renal artery stenting compromised the renal blood perfusion, resulting in serious impairment of renal function, which was the indication for re-intervention. However, it was difficult to identify the reasons for lumen narrowing on performing US, CTA, and RAG. IVUS could provide a clear diagnosis of renal artery IMH and help determine the treatment. To prevent haematoma extending to the distal end, the important strategy was that the renal artery dilatation was carried out from the distal to the proximal end. The diameter of the compressed lumen subsequently improved. However, the residual stenosis of the lumen remained more than 50%. To avoid re-enlargement of haematoma, we implanted the second stent. Subsequently, the blood flow was satisfactory, and the distal segment of the lumen was unobstructed. If RAG and IVUS had not been performed, this case would have been misdiagnosed as dissection or thrombosis of the renal artery, leading to incorrect treatment and further aggravation of the renal injury.

To our knowledge, this is the first case report of the rare complication of subacute renal artery IMH after successful renal artery stenting. Owing to the diagnosis and treatment under the guidance of IVUS, the clinical outcomes were satisfactory.

## Supplementary material

Supplementary material is available at *European Heart Journal - Case Reports* online.

**Slide sets:** A fully edited slide set detailing this case and suitable for local presentation is available online as Supplementary data.

**Consent:** The authors confirm that written consent for submission and publication of this case report including image(s) and associated text has been obtained from the patient in line with COPE guidance.

**Conflict of interest:** none declared.

## Supplementary Material

ytz014_Supplementary_Video
