# Supplementary material for: Rare complication of subacute renal artery intramural haematoma after renal artery stenting: a case report
Source: Eur Heart J Case Rep. 2019 Apr 8;3(2):ytz014. doi: 10.1093/ehjcr/ytz014 (PMC6601150; doi:10.1093/ehjcr/ytz014)
Supplement: ytz014_Supplementary_Video [file ytz014_supplementary_video.zip › ytz014_suppl_data/ytz014_Slide_Set.pptx]

## Slide 1
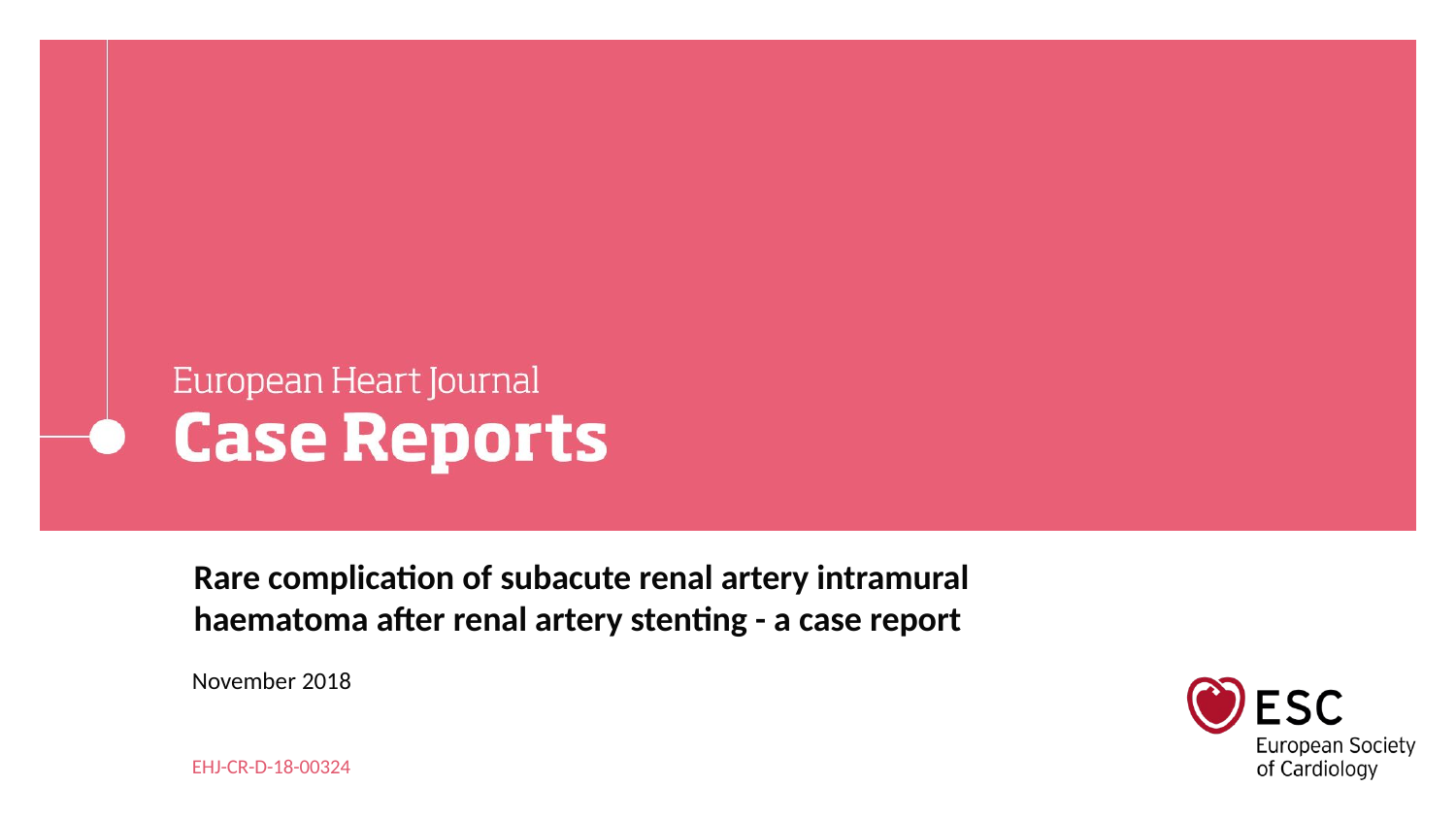

# Rare complication of subacute renal artery intramural haematoma after renal artery stenting - a case report
November 2018
EHJ-CR-D-18-00324

## Slide 2
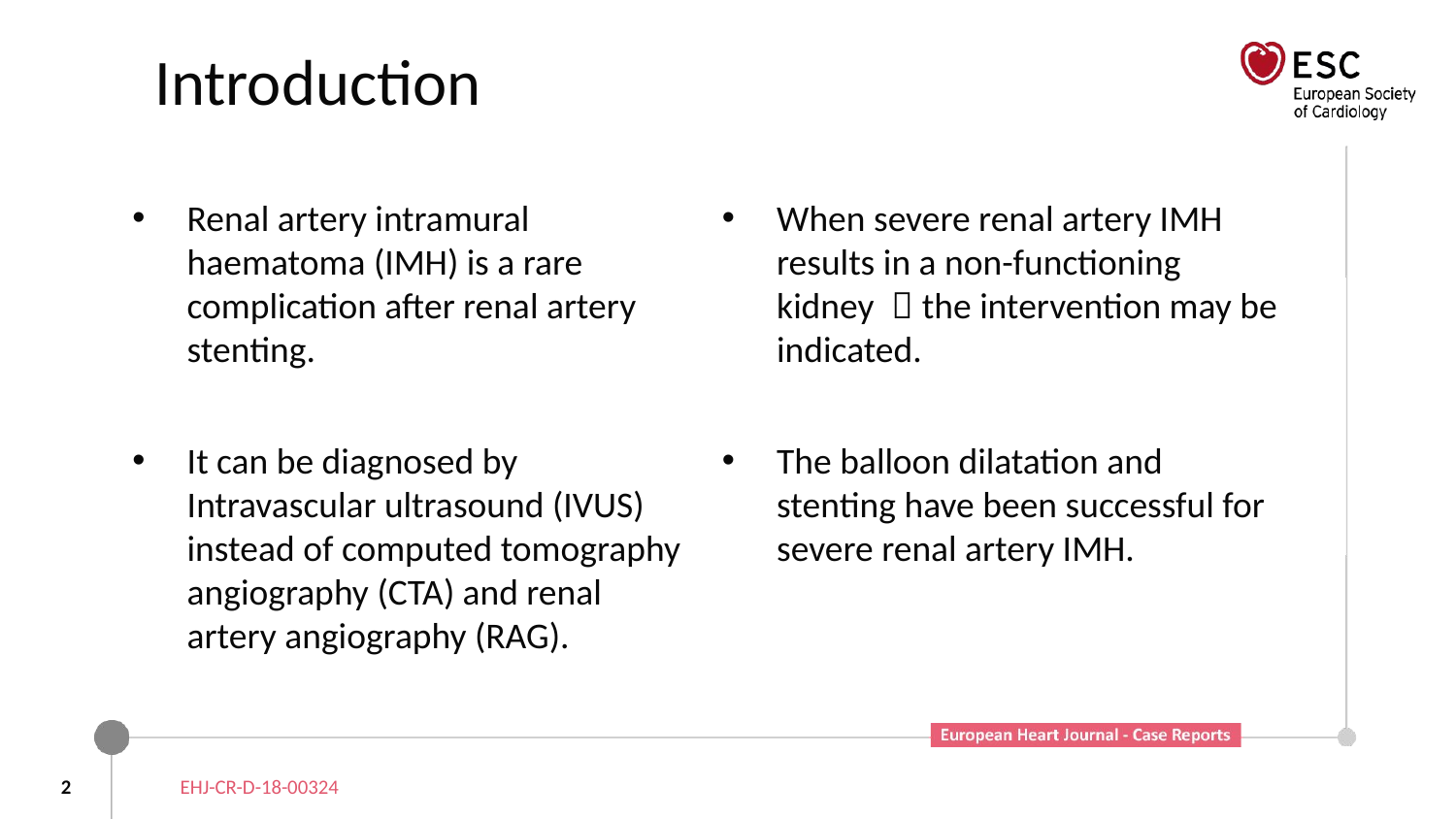

# Introduction
Renal artery intramural haematoma (IMH) is a rare complication after renal artery stenting.
It can be diagnosed by Intravascular ultrasound (IVUS) instead of computed tomography angiography (CTA) and renal artery angiography (RAG).
When severe renal artery IMH results in a non-functioning kidney ，the intervention may be indicated.
The balloon dilatation and stenting have been successful for severe renal artery IMH.
EHJ-CR-D-18-00324
2

## Slide 3
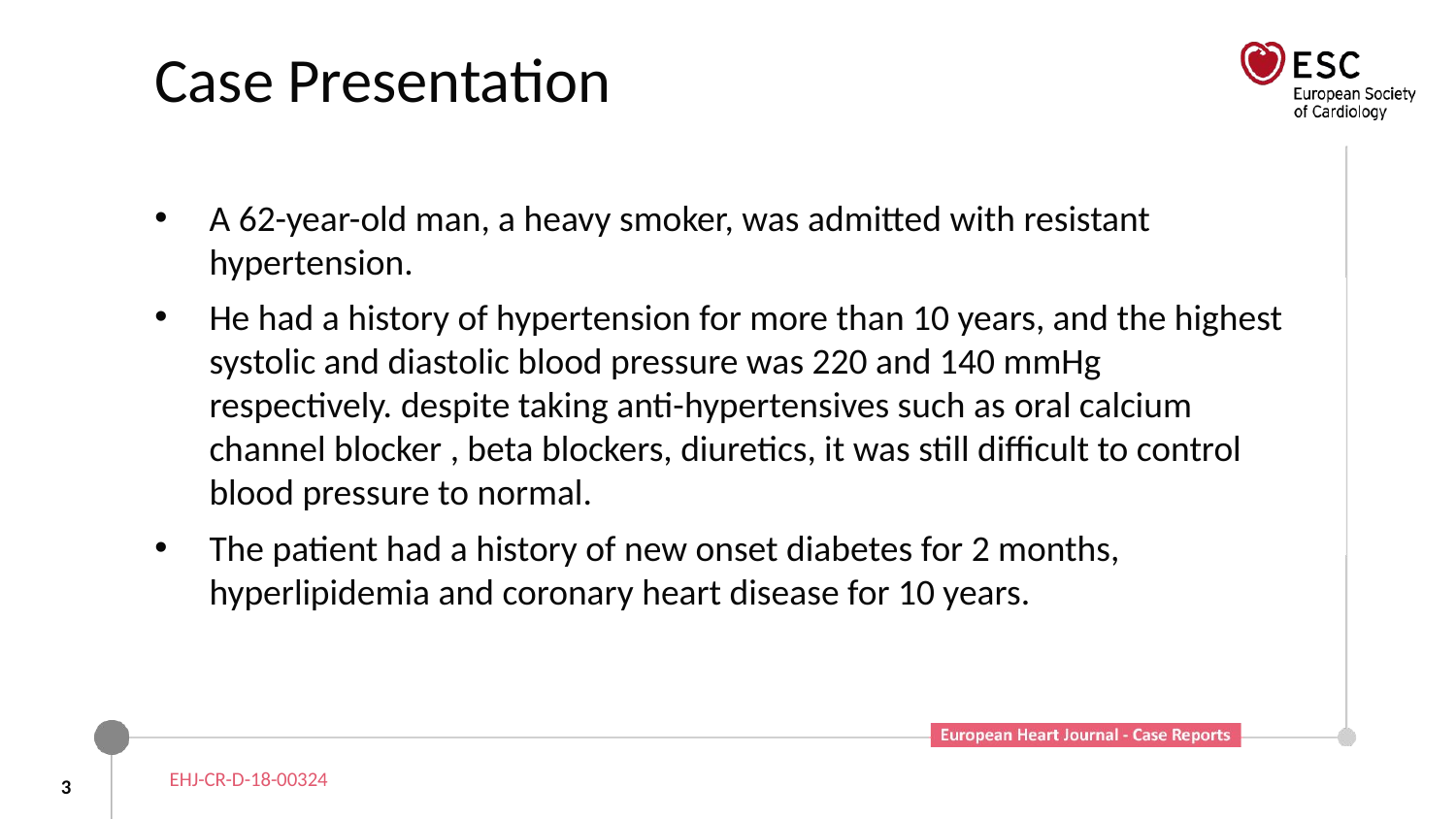

# Case Presentation
A 62-year-old man, a heavy smoker, was admitted with resistant hypertension.
He had a history of hypertension for more than 10 years, and the highest systolic and diastolic blood pressure was 220 and 140 mmHg respectively. despite taking anti-hypertensives such as oral calcium channel blocker , beta blockers, diuretics, it was still difficult to control blood pressure to normal.
The patient had a history of new onset diabetes for 2 months, hyperlipidemia and coronary heart disease for 10 years.
EHJ-CR-D-18-00324
3

## Slide 4
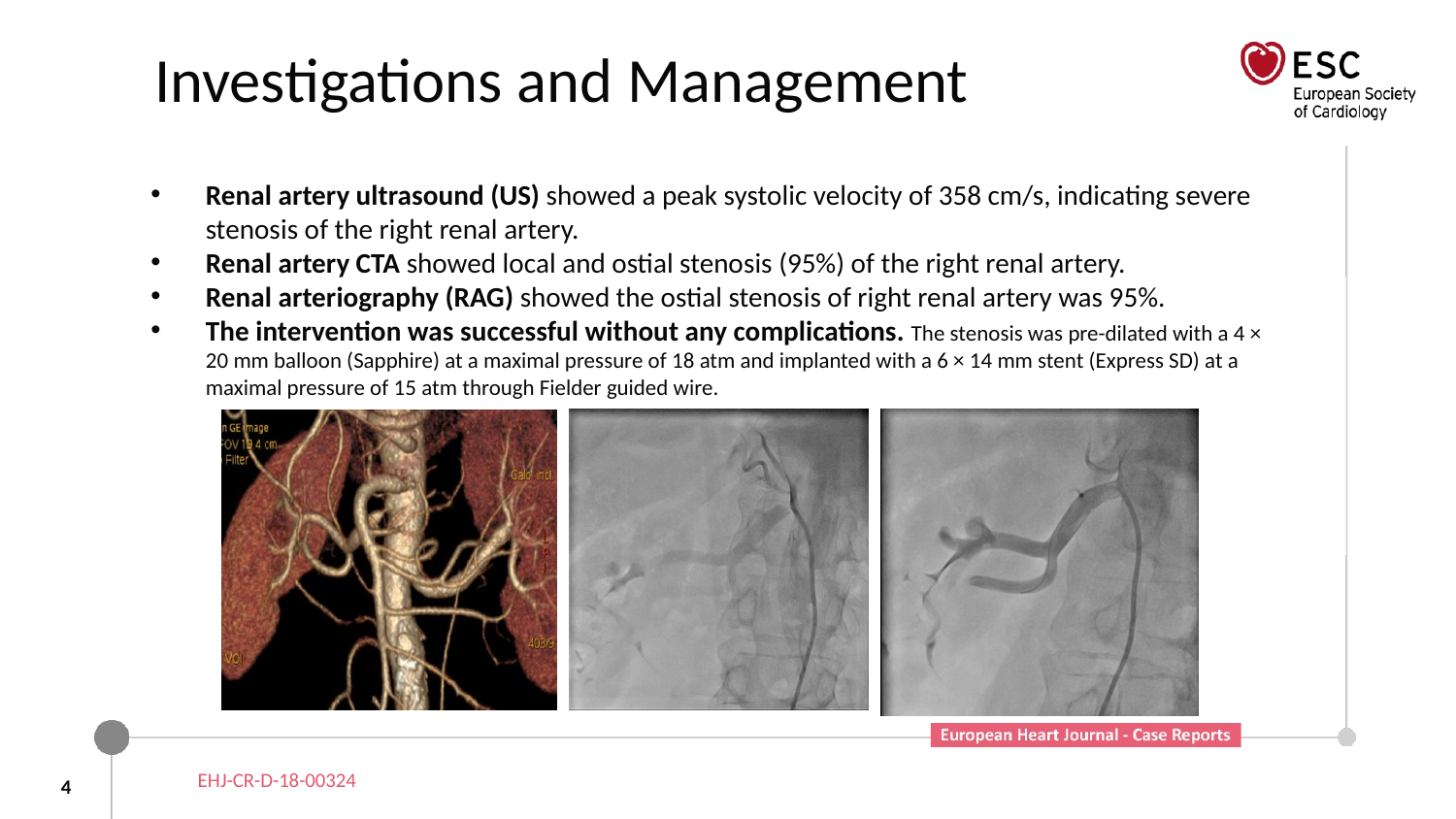

# Investigations and Management
Renal artery ultrasound (US) showed a peak systolic velocity of 358 cm/s, indicating severe stenosis of the right renal artery.
Renal artery CTA showed local and ostial stenosis (95%) of the right renal artery.
Renal arteriography (RAG) showed the ostial stenosis of right renal artery was 95%.
The intervention was successful without any complications. The stenosis was pre-dilated with a 4 × 20 mm balloon (Sapphire) at a maximal pressure of 18 atm and implanted with a 6 × 14 mm stent (Express SD) at a maximal pressure of 15 atm through Fielder guided wire.
EHJ-CR-D-18-00324
4

## Slide 5
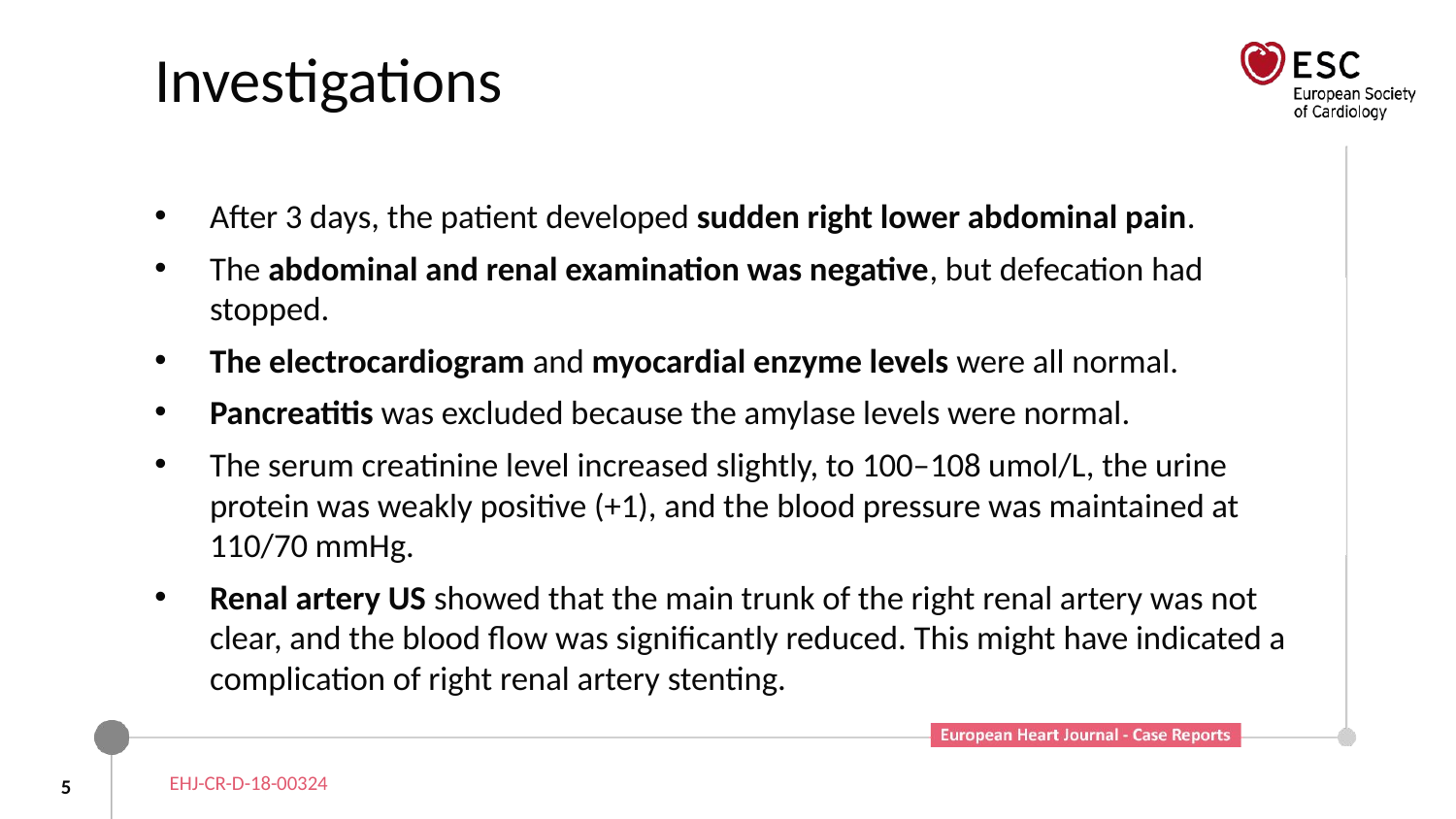

# Investigations
After 3 days, the patient developed sudden right lower abdominal pain.
The abdominal and renal examination was negative, but defecation had stopped.
The electrocardiogram and myocardial enzyme levels were all normal.
Pancreatitis was excluded because the amylase levels were normal.
The serum creatinine level increased slightly, to 100–108 umol/L, the urine protein was weakly positive (+1), and the blood pressure was maintained at 110/70 mmHg.
Renal artery US showed that the main trunk of the right renal artery was not clear, and the blood flow was significantly reduced. This might have indicated a complication of right renal artery stenting.
EHJ-CR-D-18-00324
5

## Slide 6
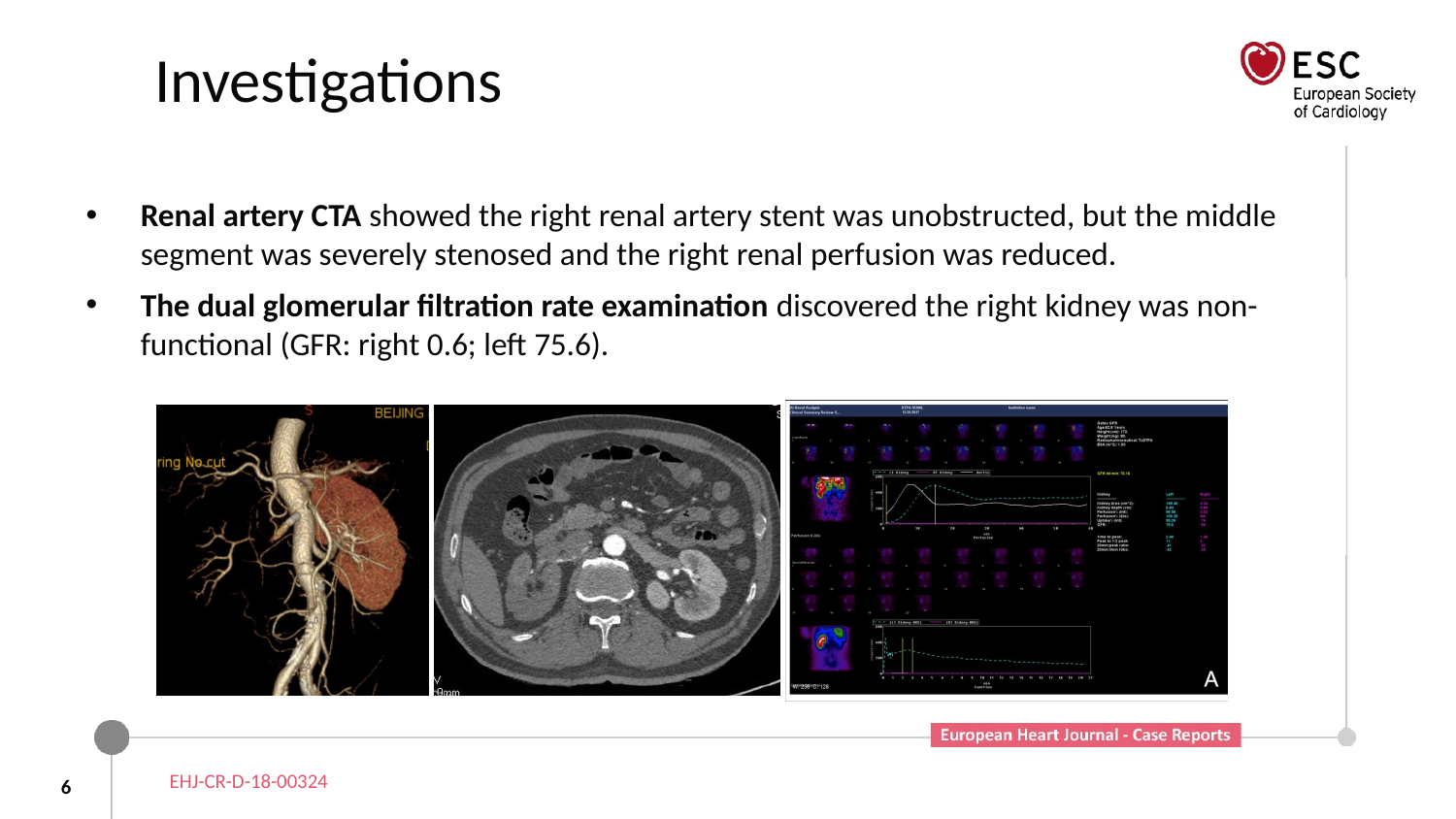

# Investigations
Renal artery CTA showed the right renal artery stent was unobstructed, but the middle segment was severely stenosed and the right renal perfusion was reduced.
The dual glomerular filtration rate examination discovered the right kidney was non-functional (GFR: right 0.6; left 75.6).
EHJ-CR-D-18-00324
6

## Slide 7
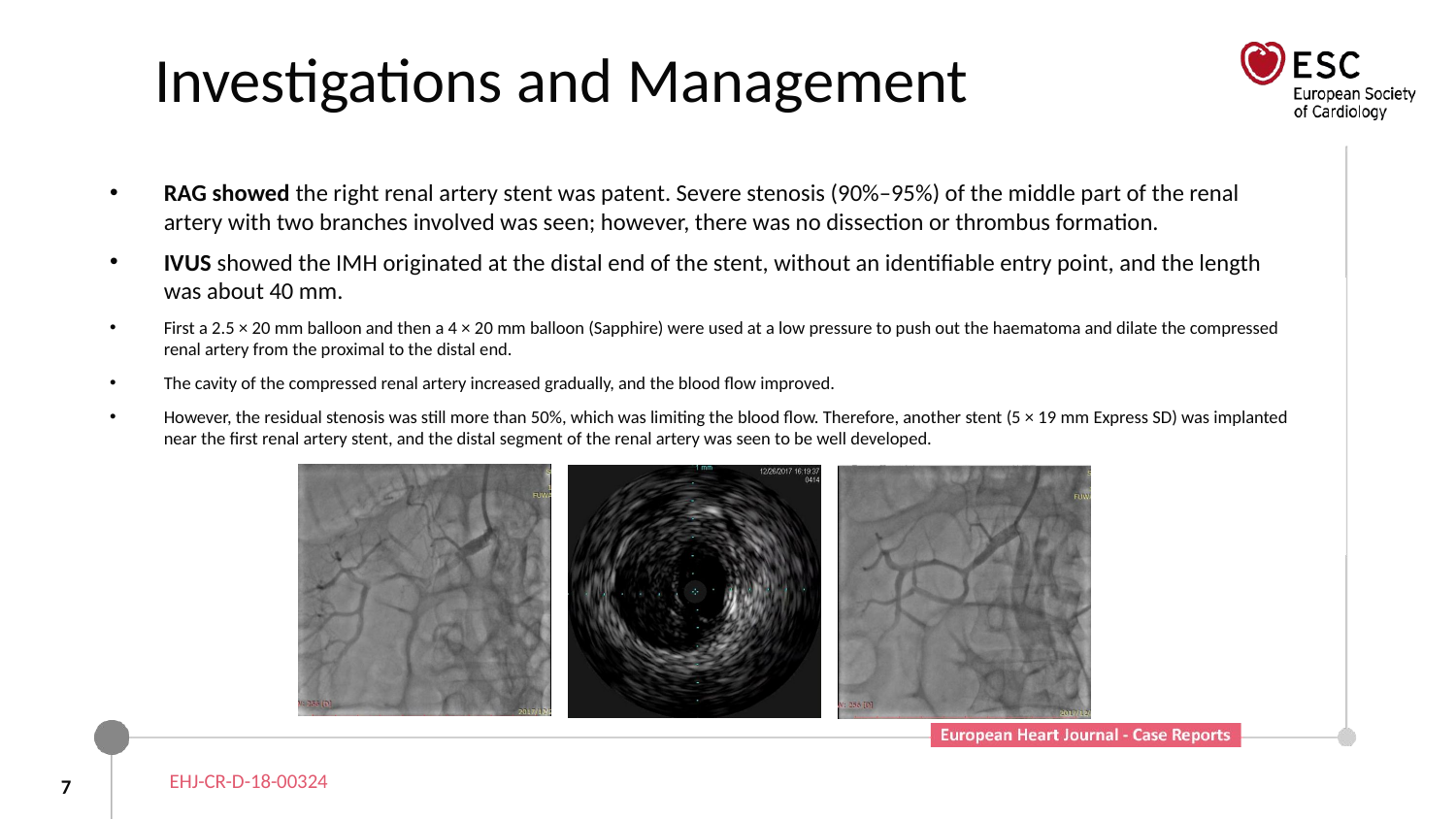

# Investigations and Management
RAG showed the right renal artery stent was patent. Severe stenosis (90%–95%) of the middle part of the renal artery with two branches involved was seen; however, there was no dissection or thrombus formation.
IVUS showed the IMH originated at the distal end of the stent, without an identifiable entry point, and the length was about 40 mm.
First a 2.5 × 20 mm balloon and then a 4 × 20 mm balloon (Sapphire) were used at a low pressure to push out the haematoma and dilate the compressed renal artery from the proximal to the distal end.
The cavity of the compressed renal artery increased gradually, and the blood flow improved.
However, the residual stenosis was still more than 50%, which was limiting the blood flow. Therefore, another stent (5 × 19 mm Express SD) was implanted near the first renal artery stent, and the distal segment of the renal artery was seen to be well developed.
EHJ-CR-D-18-00324
7

## Slide 8
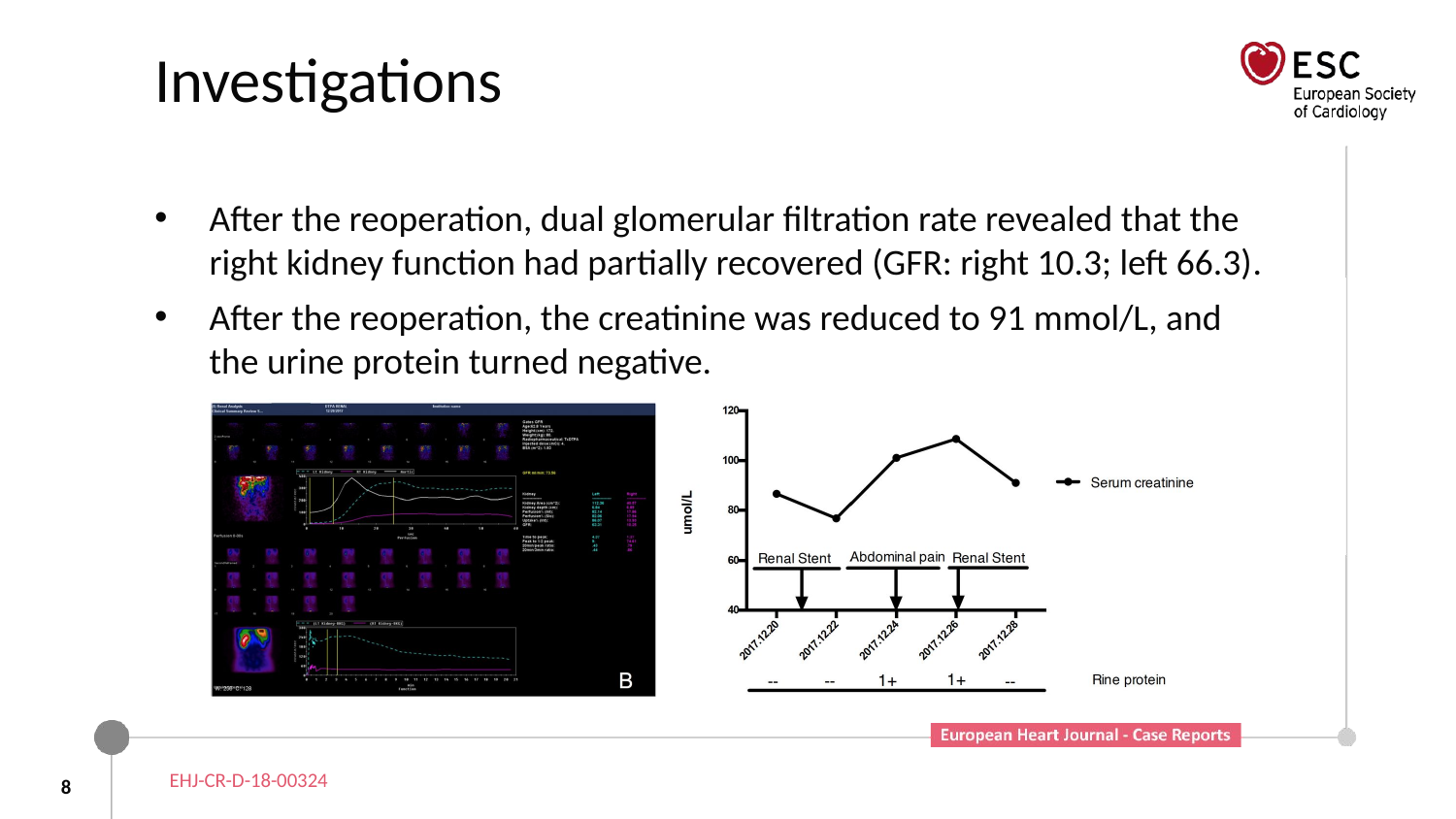

# Investigations
After the reoperation, dual glomerular filtration rate revealed that the right kidney function had partially recovered (GFR: right 10.3; left 66.3).
After the reoperation, the creatinine was reduced to 91 mmol/L, and the urine protein turned negative.
EHJ-CR-D-18-00324
8

## Slide 9
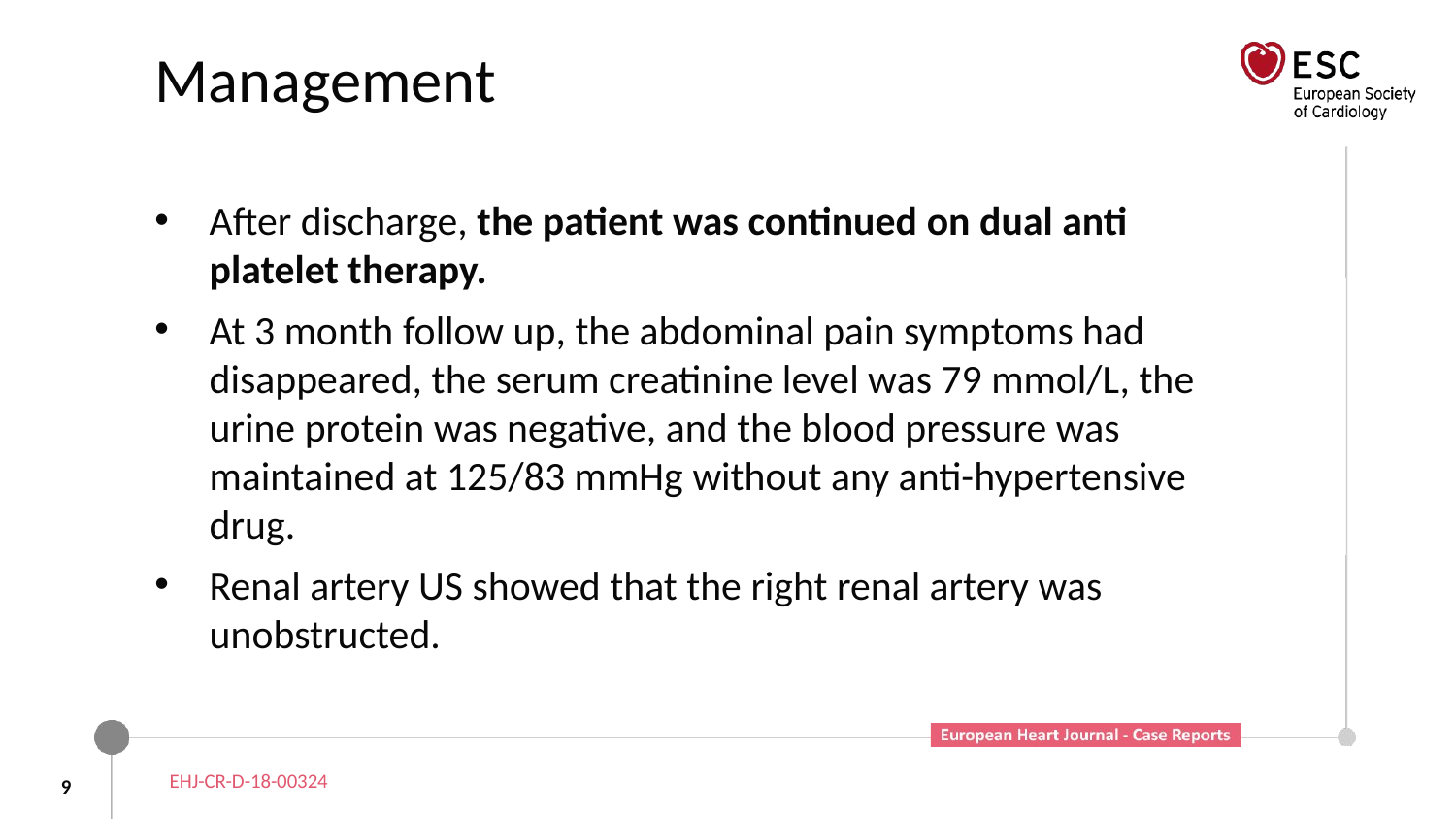

# Management
After discharge, the patient was continued on dual anti platelet therapy.
At 3 month follow up, the abdominal pain symptoms had disappeared, the serum creatinine level was 79 mmol/L, the urine protein was negative, and the blood pressure was maintained at 125/83 mmHg without any anti-hypertensive drug.
Renal artery US showed that the right renal artery was unobstructed.
EHJ-CR-D-18-00324
9

## Slide 10
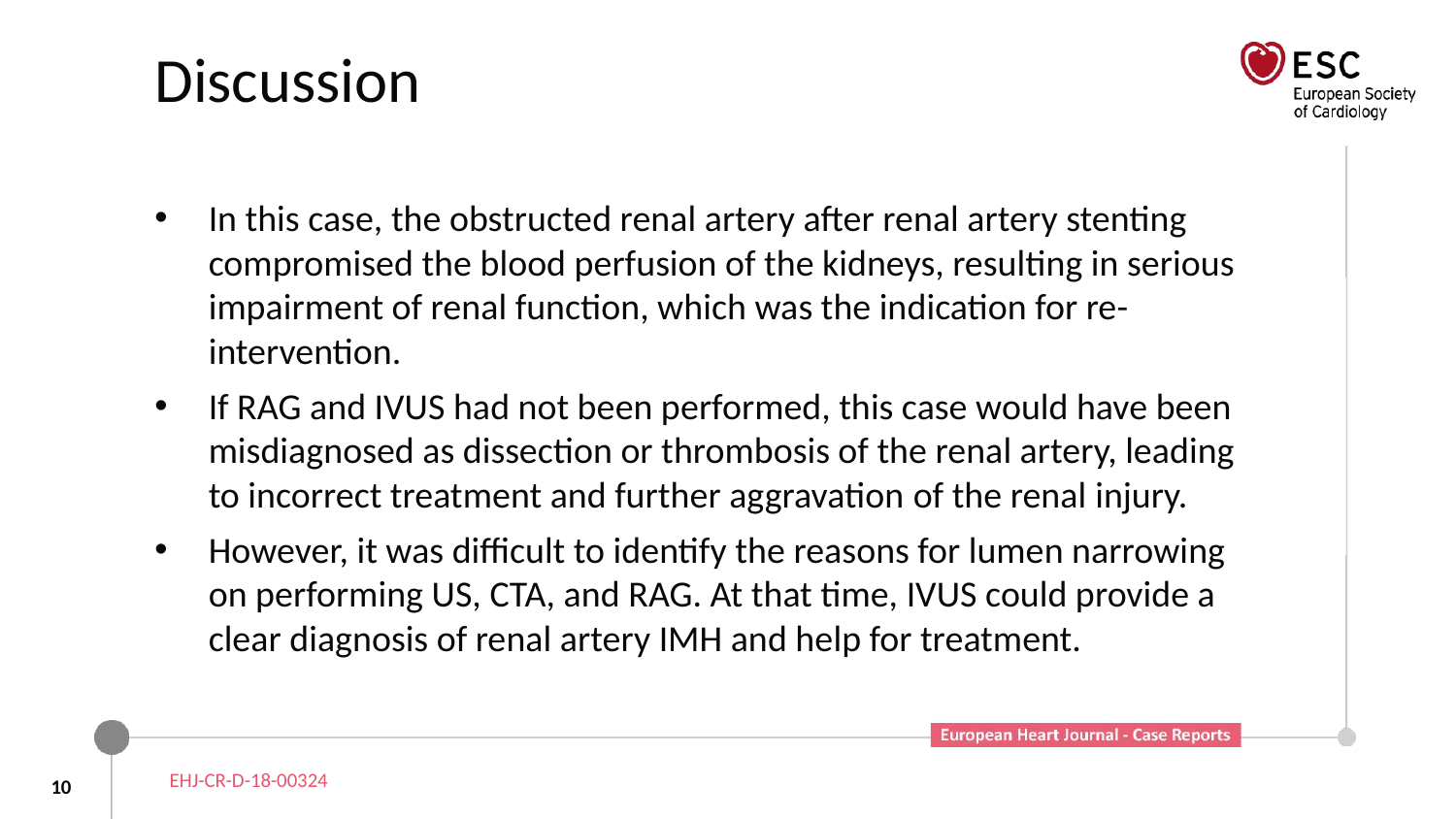

# Discussion
In this case, the obstructed renal artery after renal artery stenting compromised the blood perfusion of the kidneys, resulting in serious impairment of renal function, which was the indication for re-intervention.
If RAG and IVUS had not been performed, this case would have been misdiagnosed as dissection or thrombosis of the renal artery, leading to incorrect treatment and further aggravation of the renal injury.
However, it was difficult to identify the reasons for lumen narrowing on performing US, CTA, and RAG. At that time, IVUS could provide a clear diagnosis of renal artery IMH and help for treatment.
EHJ-CR-D-18-00324
10

## Slide 11
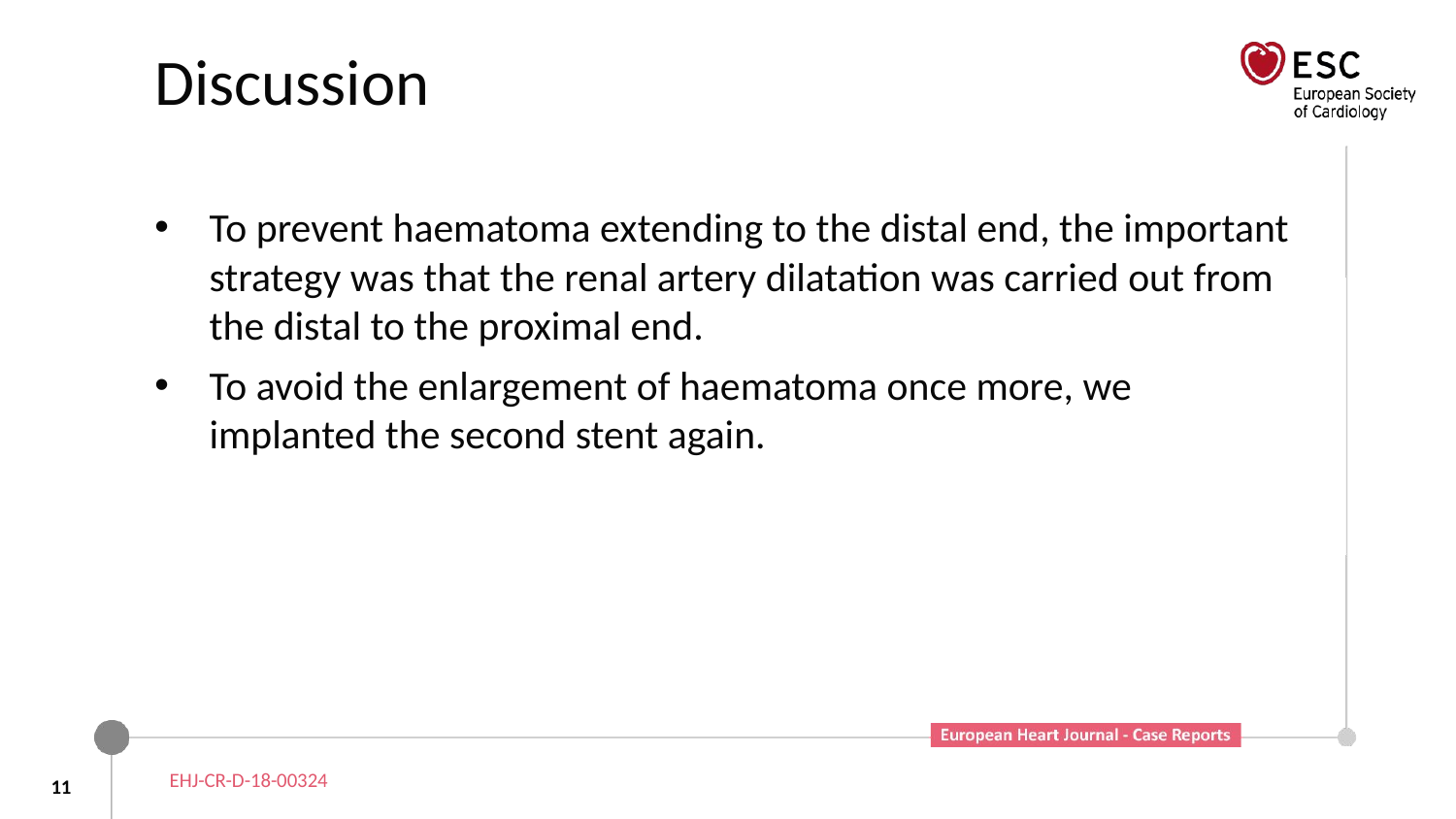

# Discussion
To prevent haematoma extending to the distal end, the important strategy was that the renal artery dilatation was carried out from the distal to the proximal end.
To avoid the enlargement of haematoma once more, we implanted the second stent again.
EHJ-CR-D-18-00324
11

## Slide 12
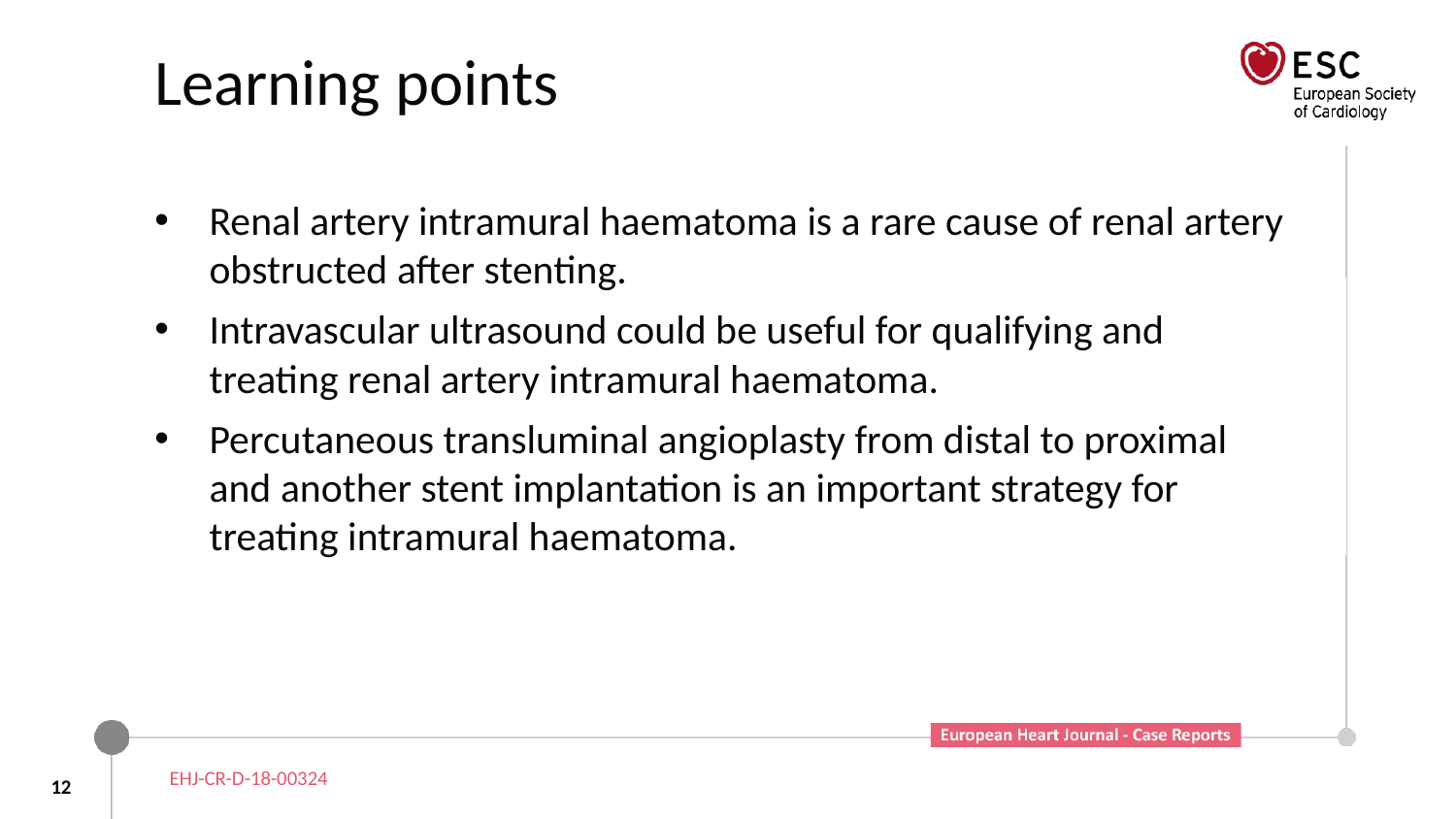

# Learning points
Renal artery intramural haematoma is a rare cause of renal artery obstructed after stenting.
Intravascular ultrasound could be useful for qualifying and treating renal artery intramural haematoma.
Percutaneous transluminal angioplasty from distal to proximal and another stent implantation is an important strategy for treating intramural haematoma.
EHJ-CR-D-18-00324
12

## Slide 13
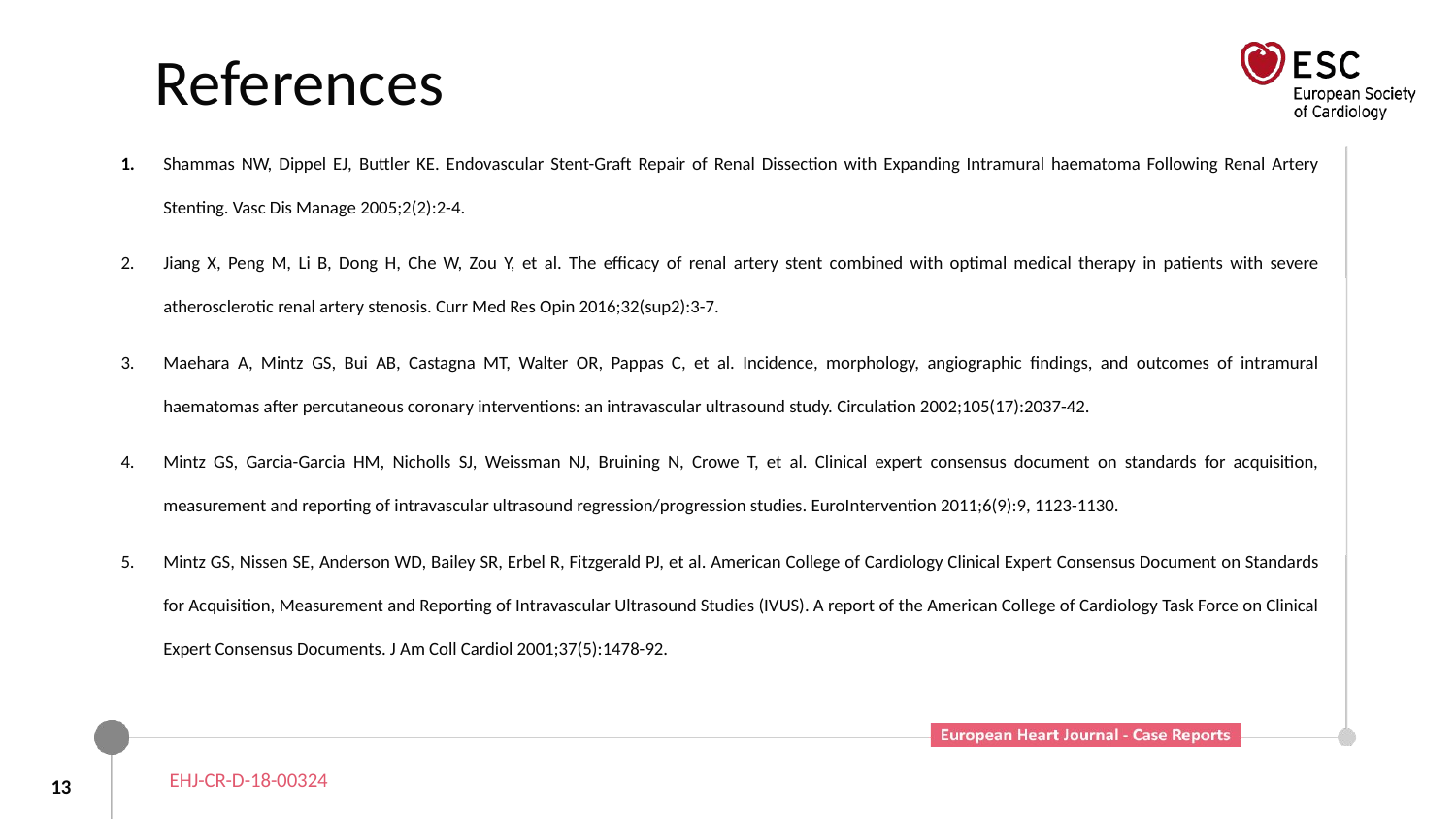

# References
1.	Shammas NW, Dippel EJ, Buttler KE. Endovascular Stent-Graft Repair of Renal Dissection with Expanding Intramural haematoma Following Renal Artery Stenting. Vasc Dis Manage 2005;2(2):2-4.
2.	Jiang X, Peng M, Li B, Dong H, Che W, Zou Y, et al. The efficacy of renal artery stent combined with optimal medical therapy in patients with severe atherosclerotic renal artery stenosis. Curr Med Res Opin 2016;32(sup2):3-7.
3.	Maehara A, Mintz GS, Bui AB, Castagna MT, Walter OR, Pappas C, et al. Incidence, morphology, angiographic findings, and outcomes of intramural haematomas after percutaneous coronary interventions: an intravascular ultrasound study. Circulation 2002;105(17):2037-42.
4.	Mintz GS, Garcia-Garcia HM, Nicholls SJ, Weissman NJ, Bruining N, Crowe T, et al. Clinical expert consensus document on standards for acquisition, measurement and reporting of intravascular ultrasound regression/progression studies. EuroIntervention 2011;6(9):9, 1123-1130.
5.	Mintz GS, Nissen SE, Anderson WD, Bailey SR, Erbel R, Fitzgerald PJ, et al. American College of Cardiology Clinical Expert Consensus Document on Standards for Acquisition, Measurement and Reporting of Intravascular Ultrasound Studies (IVUS). A report of the American College of Cardiology Task Force on Clinical Expert Consensus Documents. J Am Coll Cardiol 2001;37(5):1478-92.
EHJ-CR-D-18-00324
13
